# Perceptions and Motivations for Uterus Transplant in Transgender Women

**DOI:** 10.1001/jamanetworkopen.2020.34561

**Published:** 2021-01-20

**Authors:** Benjamin P. Jones, Abirami Rajamanoharan, Saaliha Vali, Nicola J. Williams, Srdjan Saso, Meen-Yau Thum, Sadaf Ghaem-Maghami, Isabel Quiroga, Cesar Diaz-Garcia, Philip Thomas, Stephen Wilkinson, Joseph Yazbek, J. Richard Smith

**Affiliations:** 1 West London Gynaecological Cancer Centre, Hammersmith Hospital, Imperial College NHS Trust, London, United Kingdom; 2 Department of Surgery and Cancer, Imperial College London, London, United Kingdom; 3 Department of Politics, Philosophy and Religion, Lancaster University, Lancaster, United Kingdom; 4 Lister Fertility Clinic, The Lister Hospital, London, United Kingdom; 5 The Oxford Transplant Centre, The Churchill Hospital, Oxford University Hospitals NHS Trust, Oxford, United Kingdom; 6 IVI London, IVIRMA Global, London, United Kingdom; 7 Department of Gender Surgery, Charing Cross Hospital, London, United Kingdom

## Abstract

**Importance:**

Uterus transplant has been demonstrated to be a viable fertility-restoring treatment for women categorized as female at birth with absolute uterine factor infertility. Recent advancements, as well as considerations of fairness and equality in reproductive care, have now led to the possibility of uterus transplant being undertaken in transgender women.

**Objective:**

To investigate the reproductive aspirations of transgender women and their perceptions of uterus transplant.

**Design, Setting, and Participants:**

This cross-sectional survey study used a 27-item electronic questionnaire to investigate the reproductive aspirations of 182 transgender women older than 16 years, including their perceptions of and motivations for uterus transplant, between May 1 and November 1, 2019.

**Main Outcomes and Measures:**

Perceptions of and motivations for uterus transplant, including perceived significance of the ability to gestate, menstruate, and have a physiologically functioning vagina.

**Results:**

A total of 182 transgender women completed the questionnaire; most women (109 [60%]) were aged 20 to 29 years. Most did not have children prior to transitioning (167 [92%]) and expressed a desire to have children in the future (171 [94%]). In addition, most respondents agreed or strongly agreed that the ability to gestate and give birth to children (171 [94%]) and menstruate (161 [88%]) would enhance perceptions of their femininity. Similarly, high proportions strongly agreed or agreed that having a transplanted, functioning vagina would improve their sexual experience (163 [90%]), improve their quality of life (163 [90%]), and help them to feel like more of a woman (168 [92%]). Nearly all respondents (180 [99%]) believed that uterus transplant would lead to greater happiness in transgender women. More than three-quarters of the respondents (140 [77%]) strongly agreed or agreed that they would be more inclined to cryopreserve sperm if uterus transplant became a realistic option.

**Conclusions and Relevance:**

This study provides insights into the reproductive aspirations of transgender women and reports on their multifaceted motivation to undergo uterus transplant. The survey responses suggest that transgender women would choose to have female physiologic experiences, such as menstruation and gestation, as well as potentially having a physiologically functioning transplanted vagina. If proven feasible and safe in this setting, uterus transplant may facilitate the achievement of reproductive aspirations, improve quality of life, and further alleviate dysphoric symptoms in transgender women.

## Key Points

**Question:**

What are the perceptions and motivations of transgender women for uterus transplant?

**Findings:**

This survey study of 182 transgender women found that to more than 90% of the respondents indicated that uterus transplant may improve quality of life in transgender women, alleviate dysphoric symptoms, and enhance feelings of femininity.

**Meaning:**

This report on the desire and willingness of transgender women to undergo uterus transplant may support the need for further animal and cadaveric model research, which is necessary to assess the feasibility of performing this procedure in transgender women.

## Teaser

This survey study assesses perceptions of and motivations for uterus transplant among transgender women and explores their willingness to undergo the procedure.

## Introduction

Gender dysphoria is defined as a persistent discomfort with one’s gender identity or biological sex. Despite an absence of formal epidemiologic evidence, gender dysphoria is estimated to affect up to 1.4% of adult males.^1^ Management of gender dysphoria in transgender women is complex and requires individualized multidisciplinary care between various medical, psychological, and surgical specialists. Treatment typically includes one or a combination of psychological input, hormonal therapy, or gender affirmation surgery.

Individual reproductive aspirations are influenced by the context in which they arise. Anatomic, physiologic, social, cultural, legal, and economic factors all play a central role in their development. For transgender women, infertility is a consequence of undergoing gender affirmation surgery. Supplemental estrogen therapy has also been shown to reduce sperm counts and motility,^2^ highlighting the adverse reproductive impact associated with physical realignment with gender identity. Fertility preservation, by cryopreserving sperm, should be offered to those desirous of biologically related offspring after medical or surgical transition,^3^ with subsequent use of donor eggs in surrogate individuals or with a future female partner.^3^ However, although approximately half of transgender women desire biologically related children in the future,^4^ fertility preservation rates remain low, owing to various reasons, such as financial barriers and unwillingness to delay transition.^5,6^ While the ability to gestate is not essential for a good quality of life, many transgender women experience significant psychological and social harms as a consequence of an inability to fulfill their own reproductive aspirations. These harms are exacerbated by social and cultural attitudes, norms, and expectations, which valorize reproduction, genetic relatedness, and parenthood.^7,8,9^ Moreover, many cisgender women report distress, depression, loss of gender identity, and an enduring sense of incompleteness and grief as a consequence of infertility.^10^

Uterus transplant has been demonstrated to be a viable fertility-restoring treatment for women categorized as female at birth with absolute uterine factor infertility (AUFI). The development of uterus transplant was primarily motivated by the potential to ameliorate unhappiness caused by a discrepancy between procreative ability and reproductive aspirations. The transplant would provide women with AUFI the opportunity to conceive, gestate, and give birth to genetically related offspring. More than 70 cases of uterus transplant have been performed worldwide, and detailed outcomes have been reported from 45 cases, including 9 live births.^11^ Rapid advancements in uterus transplant research, as well as considerations of fairness and equality in reproductive care, have now led to discussions of the possibility of uterus transplant in transgender women. Uterus transplant in transgender women seeks to align reproductive capacity with aspiration and alleviate the dysphoria that may arise as a result of being unable to attain parenthood.^12,13^ Significant additional psychological and social benefits following uterus transplant could also occur, particularly when considering that parenting has been identified as a protective factor in suicide risk for transgender women.^14^ Moreover, reproductive rights are recognized as human rights,^15^ and legislation in the UK means that it would be illegal to refuse to perform uterus transplant in transgender women on the basis of their gender identity.^16,17,18^ The UK’s Gender Recognition Act (2004) states that an individual’s gender becomes, for all purposes, the acquired gender, once a gender recognition certificate has been authorized; the Equality Act (2010) affords transgender people explicit protection from both direct and indirect discrimination related to their gender affirmation regardless of whether they have applied for or been granted a gender recognition certificate.^16,17^ Unless there is compelling justification, transgender women are entitled to the same treatment as women categorized as female at birth.

Uterus transplant for transgender women is more complex than for women categorized as female at birth and is currently a prospect with unproven feasibility requiring further research. A number of anatomic, hormonal, fertility, and obstetric issues contribute to the associated increased complexity, but no overwhelming clinical argument can be found that contradicts the feasibility of uterus transplant in this model.^18^ However, although a concern for justice and equality and to alleviate the sorrow caused by frustrated reproductive aspirations have motivated consideration of uterus transplant in this patient population, there are limited data available on perceptions of and potential demand for uterus transplant in transgender women.

The objective of this study was to investigate the reproductive aspirations of transgender women and their perceptions of uterus transplant. Data on this topic are needed to establish this population’s desire and request for uterus transplant before animal and cadaveric studies are undertaken to establish the feasibility of the procedure in this model.

## Methods

The study was initially advertised through transgender support groups, including the Beaumont Society, Translakes, SupportU, Allsorts Youth Project, Notts Trans Hub, Northern Concord, Transliving, Gendered Intelligence, and Press for Change. The study invitation was distributed through a variety of platforms, including websites, email, Facebook, Twitter, and Reddit. The only inclusion criterion was being a transgender woman older than 16 years. A consent form and an information leaflet, detailing the study and the proposed process of uterus transplant in the context of transgender women (eAppendix 1 in the Supplement), were sent to willing participants. Following receipt of the signed consent form, an electronic questionnaire was sent to all participants. The questionnaire consisted of 27 items (eAppendix 2 in the Supplement) and was distributed via email through SurveyMonkey over a 6-month period between May 1 and November 1, 2019. The survey items recorded demographic information, background information on the participants’ dysphoric symptoms and gender affirmation, and any previous attempts at fertility preservation. Further questions ascertained reproductive aspirations, perceptions of adoption and surrogacy, and opinions on uterus transplant. All the questions were closed, using tick boxes, with the option to include further comments if further description was warranted. Five-point Likert scales were used in questions related to perceptions. The study followed the American Association for Public Opinion Research (AAPOR) reporting guideline for survey studies. Ethical approval to undertake the study was received from Imperial College London, London, UK. The data were anonymous.

### Statistical Analysis

Descriptive statistical analysis was performed. To quantify the Likert scale responses to ascertain what appeared to be the most influential perceived factor, a weighted ranking system was used, assigning a score of 0 (not at all) to 4 (definitely) for each answer to the various influencing factors. SPSS, version 24, software (SPSS Inc) was used for analysis.

## Results

A total of 186 transgender women consented to participate in the study; of these, 182 subsequently completed the questionnaire in its entirety, resulting in a response rate of 98%. The demographics of the cohort are summarized in the Table. Most participants (109 [60%]) were between the ages of 20 and 29 years, were single (108 [59%]), and defined themselves as atheist (102 [56%]). More than half of the respondents were attracted to both men and women (104 [57%]), whereas lesser proportions were attracted exclusively to women (42 [23%]) or men (26 [14%]). Sixty-five respondents (35%) reported having experienced discomfort with their assigned gender at birth for 10 years or less, with similar proportions disclosing dysphoric symptoms for 11 to 20 years (63 [35%]) and more than 20 years (54 [30%]). Fifty-six respondents (31%) had received hormone therapy for less than 1 year, 79 participants (43%) had received it for 1 to 5 years, 15 participants (8%) had received it for 6 to 10 years, and 6 participants (3%) had received it for more than 10 years. Twenty-six women (14%) had not previously used hormone therapy. Twenty participants (11%) had undergone gender affirmation surgery, 15 (75%) within the past 5 years.

**Table.** Demographic Information of Male to Female Transgender Women Who Completed the Questionnaire

Variable	No. (%)
Age, y	
16-19	24 (13)
20-29	109 (60)
30-39	41 (23)
40-49	5 (3)
≥50	3 (2)
Religion	
Christianity	20 (11)
Hinduism	1 (0.5)
Atheist	102 (56)
Other	45 (25)
Would rather not say	14 (8)
Sexual orientation	
Attracted to women	42 (23)
Attracted to men	26 (14)
Attracted to both women and men	104 (57)
Would rather not say	10 (5)
Relationship status	
Single	108 (59)
Living with partner	35 (19)
Married	21 (12)
Divorced	5 (3)
Separated	1 (0.5)
Widowed	1 (0.5)
Would rather not say	11 (6)
No. of children	
Before transitioning	
0	167 (92)
1	9 (5)
2	5 (3)
3	0
≥4	1 (0.5)
After transitioning	
0	181 (99)
1	1 (0.5)

A total of 167 participants (92%) did not have children before transitioning, 9 women (5%) had 1 child, and 6 women (3%) had 2 or more children. Nearly all of the cohort (171 [94%]) expressed a desire to have children in the future. Of the 15 women (8%) who had children before transitioning, 14 (93%) indicated that they wanted further children. Only 1 woman reported having a child since transitioning. Half of the cohort (90 [50%]) strongly agreed or agreed that the current options of adoption or surrogacy are suitable methods for transgender women to have children. Of the 55 individuals (30%) who disagreed or strongly disagreed, 24 (44%) indicated that these options did not enable them to gestate. Approximately three-quarters of the cohort stated that transgender women were discriminated against compared with cisgender women in the process of adoption (144 [79%]) and surrogacy (128 [70%]).

**Figure 1.**
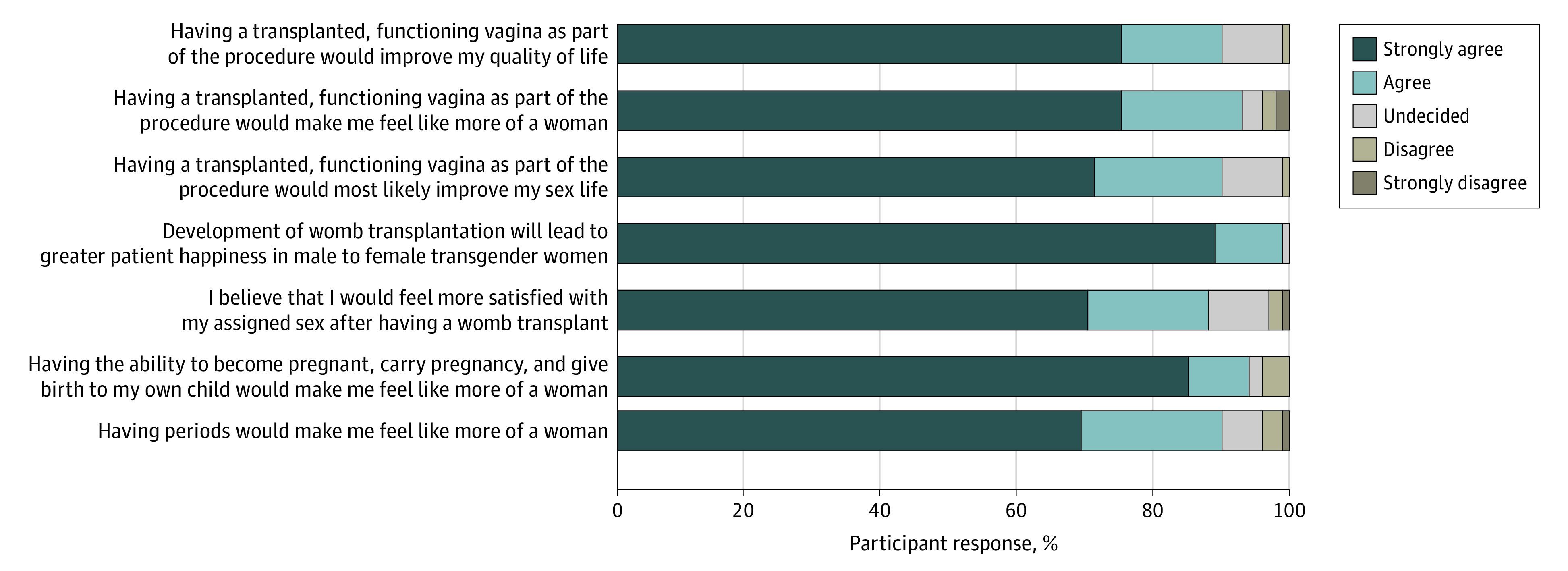
Contributing Factors That Influence Motivation and Desire to Undergo Uterine Transplantation in Transgender Women

**Figure 2.**
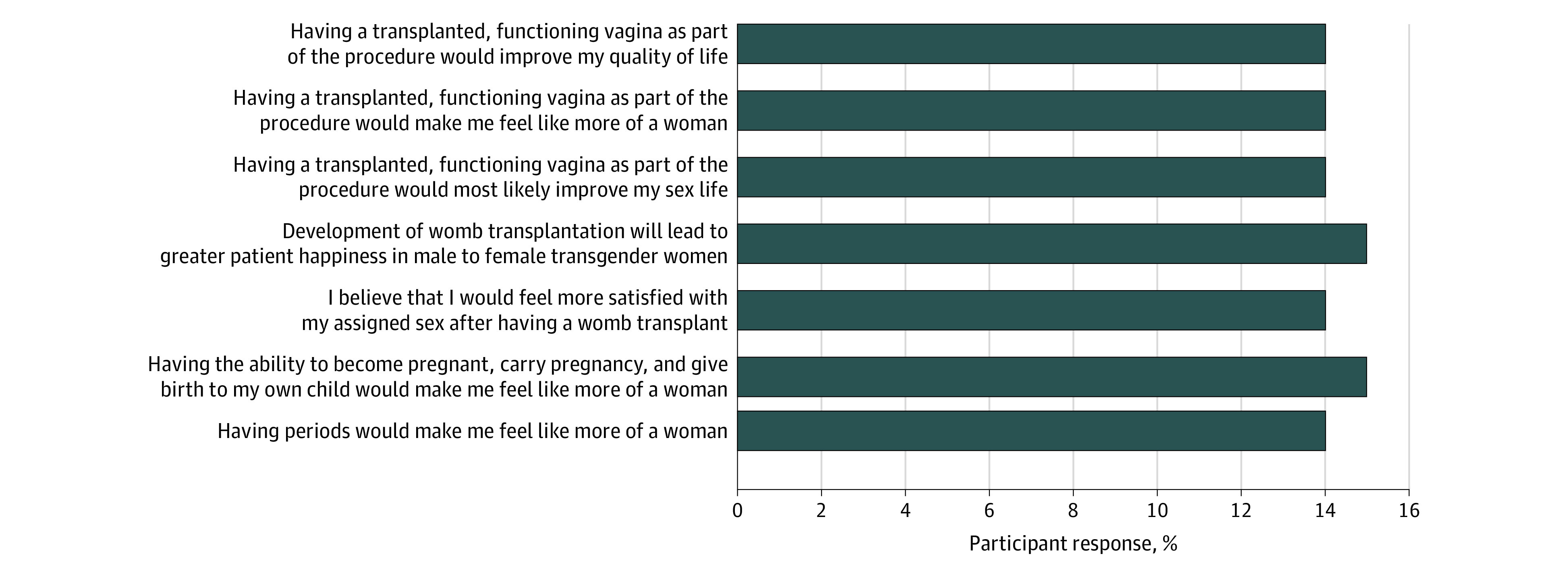
Perceived Importance of Each Influencing Factor Quantified From Likert Responses Using Weighted Scoring System

Approximately half (105 [58%]) of the respondents indicated they knew a lot or a fair amount about uterus transplant, and 74 women (41%) had heard it discussed before. A total of 177 respondents (97%) strongly agreed or agreed that they understood the benefits, and 160 participants (89%) strongly agreed or agreed that they understood the risks. Nearly all of the women (173 [95%]) believed the associated risks of uterus transplant were outweighed by its potential benefits. The factors perceived to influence their desire for uterus transplant are shown in Figure 1. Most of the women (171 [94%]) agreed or strongly agreed that the ability to become pregnant and give birth would make them feel like more of a woman. A similar proportion (161 [88%]) expressed agreement or strong agreement regarding the potential impact of having the ability to menstruate. In addition, 160 women (88%) expected to feel greater satisfaction with their gender identity after uterus transplant. Similarly high proportions of participants strongly agreed or agreed that having a transplanted, functioning vagina as part of uterus transplant would improve their sex life (163 [90%]), improve their quality of life (163 [90%]), and enable them to feel like more of a woman (168 [92%]). Using the weighted scoring system, Figure 2 presents the relative proportion of each influencing factor, with all representing similar weightings of 14% to 15%.

Nearly all respondents (180 [99%]) believed uterus transplant would lead to greater happiness in transgender women, and the same proportion (181 [99%]) indicated that if uterus transplant was feasible for women categorized as female at birth who do not have a uterus, it should be offered to transgender women. Of 156 women who reported being sexually attracted to women, 124 (79%) strongly agreed or agreed that, despite having a partner with a functioning uterus, they would want to undergo uterus transplant; 27 women (17%) were undecided, and 5 women (3%) disagreed or strongly disagreed. When considering the long-term implications of uterus transplant, 112 participants (62%) strongly agreed or agreed that they would undergo hysterectomy after completing their family to avoid the long-term risks of immunosuppressants. However, 55 women (30%) remained undecided and 15 (8%) disagreed or strongly disagreed that they would eventually undergo hysterectomy. All those who disagreed elaborated further that their unwillingness would be because of perceived potential worsening of dysphoric symptoms following hysterectomy.

**Figure 3.**
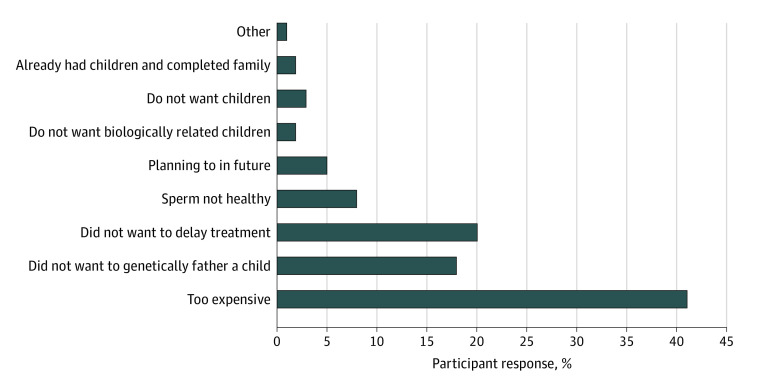
Factors in the Decision Not to Undergo Sperm Cryopreservation

Of the 182 women in our cohort, 102 (56%) were offered sperm cryopreservation, and 40 (22%) had previously cryopreserved sperm for fertility preservation purposes. Of the 142 who did not have cryopreserved sperm, the most commonly cited reasons for not doing so included financial implications (52 [37%]), not wanting to delay hormonal/surgical treatment (25 [18%]), and lack of desire to genetically father a child (23 [16%]). Figure 3 summarizes the reasons this cohort provided for not cryopreserving their sperm. Since storage of the sperm, 1 person returned to use their cryopreserved sperm with their partner, which has resulted in the birth of 2 children. When asked if they would be more inclined to cryopreserve sperm if uterus transplant became a realistic treatment option for transgender women, 140 cohort participants (77%) strongly agreed or agreed, 21 (12%) were undecided, and 21 (12%) strongly disagreed or disagreed.

## Discussion

The data presented herein report the views of transgender women on their reproductive aspirations, motivations, and desire to undergo uterus transplant should it eventually be proven feasible. These findings are comparable with those regarding the perceptions of women categorized as female at birth. For example, 95% of respondents in a UK study exploring the attitudes of women with AUFI toward uterus transplant stated that, despite the additional risks posed, they would choose uterus transplant over surrogacy and adoption.^19^ Another study, specifically assessing perceptions in 50 women with Mayer-Rokitansky-Küster-Hauser syndrome, reported that 31 of the participants (62%) were motivated to undergo uterus transplant, even after becoming aware of the associated risks involved.^20^ The results of a questionnaire answered by 60 women with AUFI in France indicated that 58% would partake in a clinical trial on uterus transplant.^21^

In our cohort, less than 10% of the women had children before transitioning. This percentage is lower than reported in previous studies undertaken between 2002 and 2008 where 22% to 64% of transgender women had children before transition.^4,22,23,24,25^ Conversely, 95% of our cohort reported a desire to have children in the future, which is higher than in a previous study in which approximately half of the respondents reported a desire for parenthood.^4^ These differences can be explained by the fact that respondents to our survey were younger, with 73% aged between 16 and 30 years and 27% aged 30 to 49 years, as opposed to the respondents in a previous study in which 70% of the participants were aged between 30 and 50 years.^4^ The younger age of women in our more recent study epitomizes the clinical implications associated with reports of earlier presentation of individuals for gender dysphoria, which is supported by the ongoing increase in the number of adolescents categorized as male at birth being referred to gender identity services.^26,27^

This shift of focus to reproduction after rather than before transition highlights the importance of fertility preservation counseling and the offer of sperm cryopreservation before medical or surgical intervention, as recommended by the World Professional Association of Transgender Health and the American Society for Reproductive Medicine.^28,29^ However, fertility preservation uptake remains low, with only around a fifth of our cohort using this opportunity. Fertility preservation among transgender youth has been reported to be consistently low, with rates varying between 1% and 38%.^5,6,30^ Barriers identified include apprehension regarding invasive procedures, financial implications, lack of awareness, and desire to not delay medical or surgical transition.^5,31^ In addition, almost a fifth of our cohort indicated that preserving sperm would conflict with their core female identity, with most attributing the expected worsening of dysphoria knowing they were a biological father. More than three-quarters of our cohort would, however, preserve sperm if uterus transplant became an option. This finding suggests that respondents to the survey were not overly concerned about inheritance of gender dysphoria in their offspring, despite increasing evidence of a genetic component associated with gender dysphoria.^32,33^

Despite adoption and surrogacy being the most readily accessible routes to parenthood, respondents reported feeling discriminated against in both processes, which may help explain why less than 1% of our cohort had children since transitioning. Although adoption or surrogacy may allow a proportion of transgender women to meet their reproductive aspirations, previous research suggests that gestation could play a vital role in conveying and consolidating a female identity^13^ and may therefore facilitate the alleviation of gender dysphoria in transgender women. Our findings appear to support this role of gestation, with most respondents (94%) stating that the ability to gestate would enhance their feelings of femininity. The role of gestation is further supported by our data suggesting that most respondents who are attracted to women (80%) would still consider undergoing uterus transplant if they were with a fertile female partner. These results are consistent with those from an earlier survey of 121 transgender women in which many respondents were more interested in the prospect of uterus transplant than sperm cryopreservation, holding pregnancy and childbirth to be superior to genetic relatedness.^4^

Our findings also suggest that transgender women may expect the ability to menstruate to enhance satisfaction with their desired gender after uterus transplant and anticipate improvements in perceptions of their femininity. The potential for having a functional vagina transplanted as part of the graft may also enhance sexual function and quality of life, and further optimize perceptions of femininity. This expectation raises the prospect of transgender women wishing to undergo uterus transplant primarily to relieve their dysphoric symptoms—with the uterus not intended for the sole purpose of childbirth in women categorized as female at birth. The intended ephemeral nature of uterus transplant creates a unique advantage over other organ transplants because, following completion of the recipient’s family, the graft can be removed, allowing the cessation of immunosuppressive medications. Although essential to prevent rejection when the graft is in situ, the use of immunosuppression is associated with a number of risks, including infection and cancer.^34,35^ Immunosuppression-related complications are dose and duration dependant.^36^ Therefore, minimizing the time the transplanted uterus is in place is an important means of reducing the level of risk for recipients. If uterus transplant is performed to allow women to experience menstruation and enhance perceptions of femininity, the duration of the graft would likely increase, significantly worsening its risk-benefit profile. Our participants reported that, although most (61%) would agree to hysterectomy following completion of their family, more than a third (39%) of the women were undecided or disagreed. Given that all those who disagreed indicated that hysterectomy would exacerbate their dysphoria symptoms, it is clear that a proportion, after extensive multidisciplinary counseling, could opt to keep their uterus despite the ongoing cumulative risk related to immunosuppression exposure.

The possibility of permanent uterus transplant in transgender women raises ethical concerns, as it would in any woman. The production of a favorable risk-benefit ratio is a key component of any justified medical procedure. The additional risks caused by permanent uterus transplant and life-long immunosuppression would therefore need to be considered and weighed against the likely benefits for the recipient. Thus, it may well be that, despite the additional benefits that permanent uterus transplant could provide to some transgender women, its risks are too great. Research into this possibility is necessary to further examine risk vs benefit and ensure that decisions about permanent uterus transplant are not clouded by individual or social biases.

Providing uterus transplant for transgender women would complicate an already complex procedure. Concerns surrounding the neovaginal anastomosis, differing male vs female pelvic size and shape, and hormonal variation call into question whether the procedure is feasible.^18^ In particular, the lack of physiologic functioning vaginal mucosa may prove problematic using the traditional uterus transplant surgical technique, highlighting the need for consideration and potential manipulation of the neovaginal microbiome in those found to be dysbiotic.^37^ Otherwise, as hypothesized by our team previously, transplantation of a longer vaginal cuff may enable a physiologically functioning vagina,^18^ which may offer additional advantages, such as improved sexual function and enhanced feelings of femininity.

### Strengths and Limitations

To our knowledge, this is the largest study of its kind to examine the reproductive aspirations of transgender women and assess detailed perceptions and motivations regarding uterus transplant. Our findings suggest that uterus transplant in transgender women could facilitate the achievement of their reproductive aspirations while concomitantly alleviating dysphoric symptoms, enhancing feelings of femininity, and potentially improving happiness and quality of life, despite the significant associated risks involved. The demonstration of desire and willingness to undergo uterus transplant in this population supports the need for further animal and cadaveric model research to assess the feasibility of performing uterus transplant in transgender women.

The study has limitations. Participants were self-selected, predominantly recruited via support groups and social media, meaning that the results are not extrapolatable to all transgender women. Because the questionnaire was advertised through various platforms, including websites and social media, it is not possible to know how many people were exposed to the offer of participation overall. Because the denominator is unknown, it is not possible to calculate the overall response rate. In addition, the exclusive use of self-reported data introduces the potential for bias. A further limitation is that, although the immunosuppression-related risk of cancer following uterus transplant was described in the patient information leaflet, the risks were not described in detail and were not robustly characterized.

## Conclusions

In this study, transgender women reported a desire to have physiologic experiences unique to cisgender women, such as menstruation and gestation, as well as potentially having a physiologically functioning transplanted vagina. Our findings suggest that some transgender women may believe the potential benefits of uterus transplant outweigh the significant risks with which it is associated and may improve quality of life, happiness, and dysphoric symptoms while enhancing feelings of femininity. As such, just as the desire to experience gestation and psychological sequelae spurred uterus transplant research in women categorized as female at birth with AUFI, uterus transplant in transgender women could be considered in the same light, and research should be undertaken regarding its feasibility.

## Notes

Supplement. **eAppendix 1.** Participant Information Sheet **eAppendix 2.** Questionnaire
