# Supplementary material for: Perceptions and Motivations for Uterus Transplant in Transgender Women
Source: JAMA Netw Open. 2021 Jan 20;4(1):e2034561. doi: 10.1001/jamanetworkopen.2020.34561 (PMC7818101; doi:10.1001/jamanetworkopen.2020.34561)
Supplement: Supplement. — eAppendix 1. Participant Information Sheet eAppendix 2. Questionnaire [file jamanetwopen-e2034561-s001.pdf]

Supplementary Online Content

Jones BP, Rajamanoharan A, Vali S, et al. Perceptions and motivations for uterus transplant in transgender women. *JAMA Netw Open*. 2021;4(1):e2034561. doi:10.1001/jamanetworkopen.2020.34561

eAppendix 1. Participant Information Sheet

eAppendix 2. Questionnaire

This supplementary material has been provided by the authors to give readers additional information about their work.

© 2021 Jones BP et al. *JAMA Network Open.*

.

eAppendix 1 - Participant Information Sheet

Full title: Male to female transgender women's perceptions to womb transplantation

You are being invited to take part in a research study. Before you decide, it is important for you to understand why the research is being done and what it will involve. Please take time to read the following information carefully and discuss it with others if you wish. Ask us if there is anything that is not clear or if you would like more information. Part 1 tells you the purpose of this study and what will happen to you if you take part. Part 2 gives you more detailed information about the conduct of the study. Ask us if there is anything that is not clear or if you would like more information. Take time to decide whether or not you wish to take part.

What is the purpose of this study?

This study will seek to gain a greater understanding of the desire, and therefore need, to perform womb transplantation as part of gender reassignment surgery. It includes a questionnaire that aims to assess demographic information, details of your previous history and treatment and details of previous fertility preservation opportunities. It also assesses feelings of your current options to acquire motherhood, your present knowledge of womb transplantation and ultimately your perceptions towards womb transplantation in transgender women as part of gender reassignment. This research study ultimately aims to assess the perceptions of transgender women to womb transplantation, to determine if there is a need to research this further. Further research studies are essential to prove that this is a feasible operation, before it could be considered being performed as a clinical procedure in transgender women.

Background information

The concept of womb transplantation has been investigated for the last 20 years, and has recently made the transition into clinical practice in natal women. At least 44 womb transplants have now been performed, and at least 12 livebirths have been achieved. The fertility rates in the Swedish cohort of seven women with successful womb transplants was 100%. It therefore appears that womb transplantation is a viable therapeutic option for natal women who do not have a functional womb. Following these developments, speculation has escalated regarding the possibility of performing womb transplantation in male to female transgender women, as part of gender reassignment surgery. The UK womb transplant research team hypothesises that it is an achievable operation, with modification to the surgical technique to the operation that has been performed so far in natal women.

The surgical technique so far includes the transplantation of the womb, including cervix (neck of the womb), a cuff of vagina, the surrounding connective tissues, as well as the major blood vessels that supply the womb. In the transgender model, the vagina would also require transplanting, to ensure the womb had a suitable environment to achieve and maintain pregnancy. This should also mean that the vagina functions normally. The ovaries would not be transplanted. The prostate shouldn't need to be removed but could be depending on each case. The surgical technique is demonstrated in figures 2 and 3:

© 2021 Jones, BP, et al. *JAMA Network Open.*

.

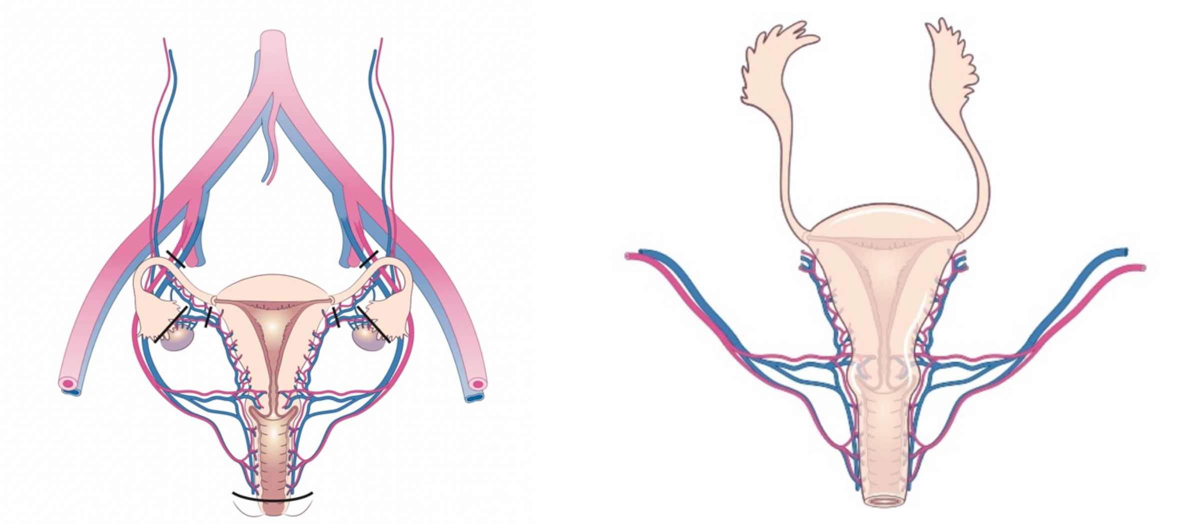

Figure 2 – *Removal of the womb, the neck of the womb (cervix), the vagina and connecting blood vessels from the donor (left) and the graft that would then be implanted into a transgender woman (right)*

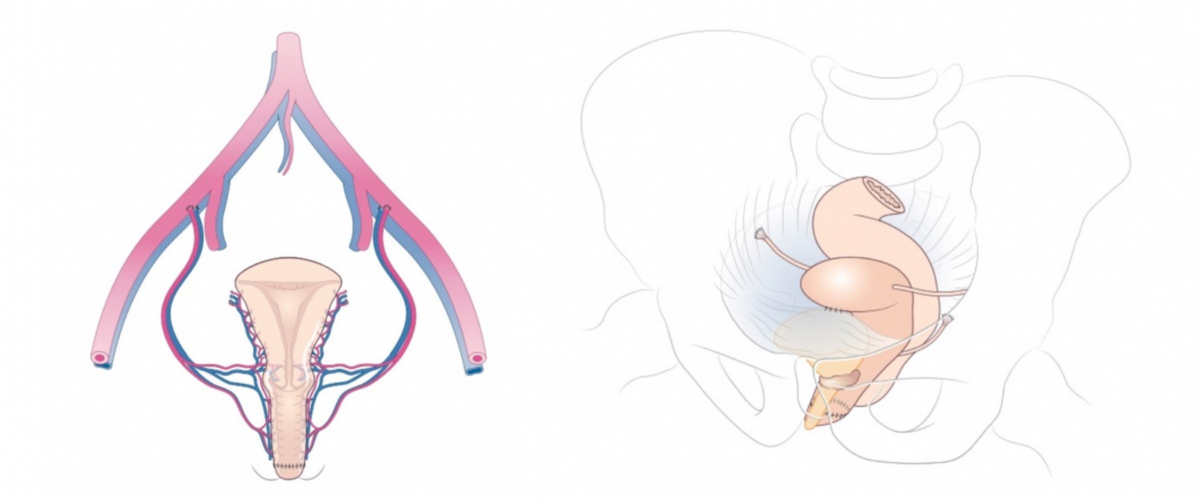

Figure 3 – *Transplantation of the womb, the neck of the womb (cervix), vagina and connecting blood vessels into the recipient (left) and the connective tissue surrounding the womb after transplantation (right)*

After the operation, the recipient would continue to take hormonal therapy, but in addition to oestrogen, progesterone would also be required to reduce the risk of abnormal cells growing in the lining of the womb. Instead of being given the same doses each day, a cyclical regime would be given instead, to enable the recipient to experience periods. This helps to determine if the womb is functional. After a period of at least 6 months, fertility treatment can be undertaken to achieve pregnancy. Embryos will have previously been created, using either the recipients stored sperm, their partners sperm or donor sperm, along with donor eggs. The pregnancy will need to be closely monitored, and will need delivery by Caesarean section. Immunosuppressive medications would need to be taken as long as the graft was in place, to reduce the risk of the body rejecting the transplanted womb. These would continue through pregnancy. These medications are deemed safe to take in pregnancy, with no increased risk of structural abnormality to the growing fetus. The long-term risks to this medication include infection and cancer, particularly if taken for longer than 5 years. As such, after the recipient has completed her family, the recipient will be advised to have the womb removed to minimise these risks.

© 2021 Jones, BP, et al. *JAMA Network Open.*

.

Why have I been chosen?

You have been invited to participate because you are a male to female transgender woman. You therefore have absolute uterine factor infertility (AUFI), a condition which affects one in 500 women of childbearing age. Women with this condition have infertility due to either the absence of their uterus (womb) or they have an anatomically or physiologically non-functioning uterus. This may be congenital, such as in transgender women, or can be acquired (for example in natal women following hysterectomy to treat cancer or following severe haemorrhage after childbirth). The causes of AUFI are shown in figure 1:

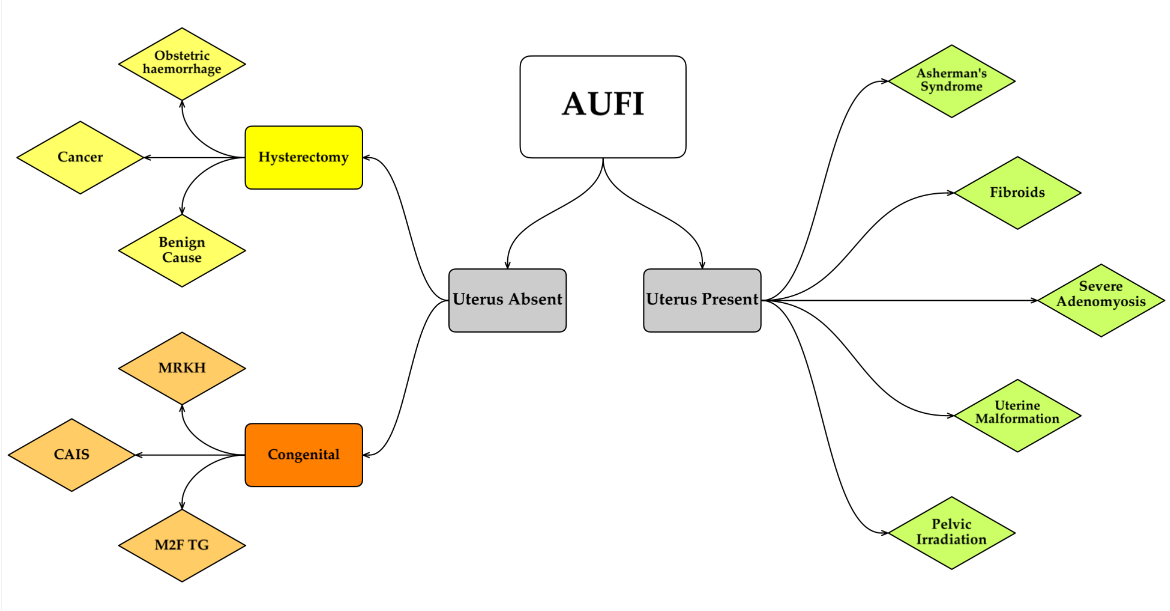

Figure 1 – *Causes of absolute uterine factor infertility (Key: AUFI; Absolute Uterine Factor Infertility, CAIS; complete androgen insensitivity syndrome, M2F TG; Male to female transgender women, MRKH; Mayer Rokitanksy Kuster Hauser Syndrome)*

Do I have to take part?

It is up to you to decide whether or not to take part. If you do decide to take part, you will be given this information sheet to keep and be asked to sign a consent form. If you decide to take part, you are still free to withdraw at any time and without giving a reason.

What do I have to do?

After reading this patient information leaflet, you will complete and sign the consent form to agree to be part of this study. Once you have scanned and returned it to Dr Benjamin Jones at Benjamin.jones@nhs.net he will send you a link to the questionnaire on www.surveymonkey.com. You will then complete the questionnaire online which contains 27 questions. It should take approximately 10 minutes to complete. Following the completion of the questionnaire, no further action will be required.

What are the possible benefits to me if I take part?

This study will give you the opportunity to express your views on womb transplantation in transgender women. Whilst the results may not directly influence you, your responses, along with the other responses received, will determine the need for our research team to pursue this research concept further.

© 2021 Jones, BP, et al. *JAMA Network Open.*

.

What are the possible disadvantages and risks of taking part?

There are no disadvantages or risks to your partaking in this questionnaire.

What if something goes wrong?

Imperial College holds a Public Liability ("negligent harm") insurance policy which applies to this trial. If you can demonstrate that you experienced serious and enduring harm as a result of your participation in this trial, you may be eligible to claim compensation without having to prove that Imperial College is at fault. If the injury resulted from any procedure which is not part of the trial, Imperial College will not be required to compensate you in this way. Your legal rights to claim compensation for injury where you can prove negligence are not affected. Please contact the Principal Investigator if you would like further information about the insurance arrangements which apply to the trial.

Will taking part be kept confidential?

This information will not identify you and will not be combined with other information in a way that could identify you. The information will only be used for the purpose of research, and cannot be used to contact you or to affect you in the future. It will not be used to make decisions about future services available to you, such as insurance.

Imperial College London is the sponsor for this study based in the United Kingdom. We will be using information from you in order to undertake this study and will act as the data controller for this study. This means that we are responsible for looking after your information and using it properly.

Information on Imperial College London's retention periods may be found at https://www.imperial.ac.uk/media/imperial-college/administration-and-support-services/records-and-archives/public/RetentionSchedule.pdf.

Legal Basis

As a university we use personally-identifiable information to conduct research to improve health, care and services. As a publicly-funded organisation, we have to ensure that it is in the public interest when we use personally-identifiable information from people who have agreed to take part in research. This means that when you agree to take part in a research study, we will use your data in the ways needed to conduct and analyse the research study. Health and care research should serve the public interest, which means that we have to demonstrate that our research serves the interests of society as a whole. We do this by following the UK Policy Framework for Health and Social Care Research.

International Transfers

There may be a requirement to transfer information to countries outside the European Economic Area (for example, to a research partner). Where this information contains your personal data, Imperial College London will ensure that it is transferred in accordance with data protection legislation. If the data is transferred to a country which is not subject to a European Commission (**EC**) adequacy decision

© 2021 Jones, BP, et al. *JAMA Network Open.*

.

in respect of its data protection standards, Imperial College London will enter into a data sharing agreement with the recipient organisation that incorporates EC approved standard contractual clauses that safeguard how your personal data is processed.

Contact us

If you wish to raise a complaint on how we have handled your personal data or if you want to find out more about how we use your information, please contact Imperial College London's Data Protection Officer via email at dpo@imperial.ac.uk, via telephone on 020 7594 3502 and via post at Imperial College London, Data Protection Officer, Faculty Building Level 4, London SW7 2AZ. If you are not satisfied with our response or believe we are processing your personal data in a way that is not lawful you can complain to the Information Commissioner's Office (ICO). The ICO does recommend that you seek to resolve matters with the data controller (us) first before involving the regulator.

Imperial College London will collect information from you and for this research study in accordance with our instructions. Imperial College London may use your name and contact details to contact you about the research study, and make sure that relevant information about the study is recorded for your care, and to oversee the quality of the study. Individuals from Imperial College London may look at your research records to check the accuracy of the research study. The only people at Imperial College London who will have access to information that identifies you will be people who need to contact you to audit the data collection process. The people who analyse the information will not be able to identify you and will not be able to find out your name or contact details. Imperial College London will keep identifiable information about you from this study for 10 years after the study has finished.

What will happen to the results of the research study?

The data collected from the study will be used to determine the need to investigate this concept further, with the final aim to perform a sustainable and achievable womb transplant operation in transgender women. The information gained from the questionnaires will contribute towards a PhD research degree for Dr Benjamin Jones. The data may also be used for publication in peer reviewed journals and included in oral and poster presentations at national and international conferences. You will not be able to be identified in any publications or presentations.

Who is organising and funding the study?

This study has been organised by the registered charity Womb Transplant UK (Charity no 1138559). No healthcare professional will receive payment for conducting this research.

Contact for further information

If you have any further questions please do not hesitate to contact the study coordinator, Dr Benjamin Jones, at Benjamin.jones@nhs.net who will be able to answer any queries.

Thank you for reading this leaflet and considering whether to take part in the study

© 2021 Jones, BP, et al. *JAMA Network Open.*

.

eAppendix 2 - Questionnaire

Full title: Male to female Transgender women's perceptions to womb transplantation

Please complete the following questionnaire after reading the participant information sheet and after you have signed the consent form.

1. **Demographic information**

a) **Age (years)**

16-19

20-29

30-39

40-49

50+

Would rather not say

b) **Assigned sex at birth**

Male

Female

c) **Currently assigned sex**

Male

Female

In process of transitioning

d) **Religion**

Christianity

Islam

Hinduism

Other

Athiest

Would rather not say

e) **Relationship status**

Single

Living with partner

Married

Divorced

© 2021 Jones, BP, et al. *JAMA Network Open.*

.

Separated

Widowed

Would rather not say

f) **Sexual orientation**

Attracted to men

Attracted to women

Attracted to both men and women

Not attracted to either men or women

Would rather not say

2. How many years ago did you develop symptoms of discomfort with the sex you were assigned at birth?

Less than 1 year

Between 1 and 5 years

Between 6 and 10 years

Between 11 and 15 years

Between 16 and 20 years

>20 years

3. If relevant, how long have you been taking hormonal therapy?

Never taken

Less than 1 year

Between 1 and 5 years

Between 6 and 10 years

Between 11 and 15 years

Between 16 and 20 years

>20 years

4. Please indicate, if relevant, how long ago you underwent gender reassignment surgery

Never had gender reassignment surgery

Less than 1 year

Between 1 and 5 years

Between 6 and 10 years

Between 11 and 15 years

Between 16 and 20 years

>20 years

© 2021 Jones, BP, et al. *JAMA Network Open.*

5. Prior to undergoing hormonal or surgical treatment, were you offered the chance to freeze your sperm for use in the future?

Yes No

6. Prior to undergoing hormonal or surgical treatment, did you freeze your sperm for future use?

Yes No

If No: What was the main reason for not preserving your sperm?

Do not want children
Do not want biologically related children
Too expensive
Sperm already not healthy after hormone therapy
Already had children and completed family
Too young and sperm not developed
Did not want to delay treatment
Other (please state)……………………………………

If Yes: Have you used the stored sperm yet?

Yes, in surrogate Yes, in partner No

7. Did you have children prior to transitioning?

Yes No

If yes: How many children did you have prior to transitioning?

0 1 2 3 4+

8. Have you had children since transitioning?

Yes No

If yes: How many

0 1 2 3 4+

9. Do you want children in the future?

Yes No

10. Do you believe that the current fertility options for women with absolute womb factor infertility (adoption and surrogacy), are suitable methods for transgender women to have children.

Strongly agree Agree Undecided Disagree Strongly Disagree

If disagree/strongly disagree, why? ………..…………………………………………

11. Do you feel that transgender women are hindered, more than natal women, by discrimination in the process of adoption?

Strongly agree Agree Undecided Disagree Strongly Disagree

If disagree/strongly disagree, why? ………..…………………………………………

© 2021 Jones, BP, et al. *JAMA Network Open.*

12. Do you feel that transgender women are hindered, more than natal women, by discrimination in the process of surrogacy?

Strongly agree Agree Undecided Disagree Strongly Disagree

If disagree/strongly disagree, why? ……….…………………………………………

13. How much do you know about Womb Transplantation?

A lot A fair amount Heard it discussed only a few times Nothing

14. I understand the benefits of Womb Transplantation.

Strongly agree Agree Undecided Disagree Strongly Disagree

If disagree/strongly disagree, why? ……….…………………………………………

15. I understand the risks of Womb Transplantation.

Strongly agree Agree Undecided Disagree Strongly Disagree

If disagree/strongly disagree, why? ……….…………………………………………

16. I believe the potential benefits of Womb Transplantation outweigh the potential risks in transgender women

Strongly agree Agree Undecided Disagree Strongly Disagree

If disagree/strongly disagree, why? ……….…………………………………………

17. I believe that having periods would make me feel like more of a woman

Strongly agree Agree Undecided Disagree Strongly Disagree

If disagree/strongly disagree, why? ……….…………………………………………

18. I believe that having the ability to become pregnant, carry pregnancy and give birth to my own child would make me feel like more of a woman

Strongly agree Agree Undecided Disagree Strongly Disagree

If disagree/strongly disagree, why? ……….…………………………………………

19. I believe that I would feel more satisfied with my assigned sex after having a womb transplant

Strongly agree Agree Undecided Disagree Strongly Disagree

If disagree/strongly disagree, why? ……….…………………………………………

20. I believe that the development of womb transplantation will lead to greater patient happiness in transgender women.

Strongly agree Agree Undecided Disagree Strongly Disagree

If disagree/strongly disagree, why? ……….…………………………………………

21. I believe that if womb transplantation becomes an established treatment for women assigned female at birth who do not have a womb, it should be offered to transgender women once it is proven to be feasible.

Strongly agree Agree Undecided Disagree Strongly Disagree

If disagree/strongly disagree, why? ……….…………………………………………

22. I believe that if womb transplantation becomes an established treatment for transgender women, I would have been more inclined to freeze my sperm to use to create embryos to be used in my womb in the future?

Strongly agree Agree Undecided Disagree Strongly Disagree

If disagree/strongly disagree, why? ……….…………………………………………

© 2021 Jones, BP, et al. *JAMA Network Open.*

.

23. As part of womb transplantation, having a transplanted, functioning vagina as part of the procedure would most likely improve my sex life

Strongly agree Agree Undecided Disagree Strongly Disagree

If disagree/strongly disagree, why? ……….…………………………………………

24. As part of womb transplantation, having a transplanted, functioning vagina as part of the procedure would make me feel like more of a woman.

Strongly agree Agree Undecided Disagree Strongly Disagree

If disagree/strongly disagree, why? ……….…………………………………………

25. As part of womb transplantation, having a transplanted, functioning vagina as part of the procedure would improve my quality of life.

Strongly agree Agree Undecided Disagree Strongly Disagree

If disagree/strongly disagree, why? ……….…………………………………………

26. If you received a womb transplant, would you be accepting of removal of the womb after you have completed your family, to avoid the long-term risks associated with the medications needed to prevent rejection?

Strongly agree Agree Undecided Disagree Strongly Disagree

If disagree/strongly disagree, why? ……….…………………………………………

27. If, when you are ready to have children, and your partner was a female and had a functioning womb, would you still want a womb transplant yourself for the purpose of having children?

Strongly agree Agree Undecided Disagree Strongly Disagree

n/a - I am sexually attracted to men

If disagree/strongly disagree, why? ……….…………………………………………

Thank you for completing this questionnaire and taking part in this study

© 2021 Jones, BP, et al. *JAMA Network Open.*
